# Physician staffed helicopter emergency medical service case identification - a before and after study in children

**DOI:** 10.1186/s13049-016-0284-6

**Published:** 2016-07-12

**Authors:** Alan A. Garner, Anna Lee, Andrew Weatherall, Mary Langcake, Zsolt J. Balogh

**Affiliations:** CareFlight, Northmead, NSW Australia; Department of Anaesthesia and Intensive Care, The Chinese University of Hong Kong, Prince of Wales Hospital, Shatin, NT Hong Kong; St George Hospital, Kogarah, NSW Australia; John Hunter Hospital, University of Newcastle, Newcastle, NSW Australia

**Keywords:** Trauma, Prehospital, Dispatch, Paediatric, Physician, HEMS

## Abstract

**Background:**

Severely injured children may have better outcomes when transported directly to a Paediatric Trauma Centre (PTC). A case identification system using the crew of a physician staffed helicopter emergency medical service (P-HEMS) that identified severely injured children for P-HEMS dispatch was previously associated with high rates of direct transfer. It was theorised that discontinuation of this system may have resulted in deterioration of system performance.

**Methods:**

Severe paediatric trauma cases were identified from a state based trauma registry over two time periods. In Period A the P-HEMS case identification system operated in parallel with a paramedic dispatcher (Rapid Launch Trauma Co-ordinator-RLTC) operating from a central control room (*n* = 71). In Period B the paramedic dispatcher operated in isolation (*n* = 126). Case identification and direct transfer rates were compared as was time to arrival at the PTC.

**Results:**

After cessation of the P-HEMS system the rate of case identification fell from 62 to 31 % (*P* < 0.001), identification of fatal cases fell from 100 to 47 % (*P* < 0.001), the rate of direct transfer to a PTC fell from 66 to 53 % (*P* = 0.076) and the time to arrival in a PTC increased from a median 69 (interquartile range 52 – 104) mins to 97 (interquartile range 56 – 305) mins (*P* = 0.003). When analysing the rate of direct transfer to a PTC as a function of team composition, after adjusting for age and injury severity scores, there was no change in the rate between the physician and paramedic groups across the two time periods (relative risk 0.92, 95 % CI: 0.44 to 1.41).

**Discussion:**

The parallel identification system improves case identification rates and decreases time to arrival at the PTC, whilst requiring RLTC authorisation preserves the safety and efficiency benefits of centralised dispatch. The model could be extended to adult patients with similar benefits.

**Conclusions:**

A case identification system relying solely on RLTC paramedics resulted in a significantly lower case identification rate and increased prehospital time with a non-significant fall in direct transfer rate to the PTC. The elimination of the P-HEMS input from the tasking system resulted in worse performance indicators and has the potential for poorer outcomes.

## Background

There is evidence that severely injured children have better outcomes if transported directly to a dedicated Paediatric Trauma Centre (PTC) [1–7]. In the State of New South Wales (NSW), Australia trauma to children 0–14 years of age accounts for only about 5 % or all severely injured patients with 192 children with an Injury Severity Score (ISS) greater than twelve occurring state wide in 2014 [8]. Prehospital triage systems therefore must be able to identify those severely injured children who would benefit from transport directly to a PTC, and dispatch a crew with the required skill set to make this determination. For those children that can be adequately managed in an Adult Trauma Centre (ATC) or non-trauma centre hospital, deployment of a physician staffed helicopter is less critical, and many can be appropriately managed by paramedic crews only.

NSW has a network of physician staffed helicopter emergency medical service (P-HEMS) operating from seven bases which collectively transport approximately 500 severely injured patients annually [8]. A previous study of the paediatric prehospital trauma system in NSW compared the performance of two case identification systems, which were operating in parallel in the Sydney region, for dispatch of a P-HEMS to severely injured children [9]. The case identification systems were:

- Direct screening of the NSW Ambulance Computer Assisted Dispatch (CAD) system (Medical Priority Dispatch System, Priority Dispatch Corp, Utah, USA) by the CareFlight P-HEMS crew via a web link
- A dedicated paramedic, the Rapid Launch Trauma Co-ordinator (RLTC), monitoring the same CAD system from a centralised control room.

This study demonstrated significant effects on the Sydney prehospital paediatric trauma system when the CareFlight P-HEMS case identification system was operating in parallel with the RLTC system with an increased likelihood that patients would be transported more rapidly and directly to a PTC. In March 2011 however, access to the CAD system by the CareFlight P-HEMS crew was withdrawn by NSW Ambulance with all tasking from that time conducted only by the RLTC in the central control centre.

This study was designed to evaluate the effect of this change in tasking systems on the Sydney paediatric trauma system. We hypothesised that the cessation of the P-HEMS screening system would result in a return to the direct PTC transfer rate that existed prior to commencement of the CareFlight P-HEMS system, and time from injury to arrival at the PTC would also therefore increase. The system change also provided an opportunity to address some limitations of the initial study. The first study was unable to determine whether cases identified by the P-HEMS system would eventually have been also identified by the RLTC as each system was not blinded to the actions of the other.

## Methods

### Aim, design and setting of the study

Ethics committee approval was obtained from the Sydney Children’s Hospitals Network Human Research Ethics Committee (LNR/15/SCHN/147). The study is a retrospective, registry-based comparison of the performance of the Sydney prehospital trauma system during two time periods. The periods are:

- Period A; 24^th^ May 2008 to 13^th^ March 2011, the Rapid Launch Trauma Coordinator (RLTC) and CareFlight P-HEMS case identification processes operated in parallel and includes the 2 year period reported in the prior study [9]. This is the entire duration of the parallel tasking model.
- Period B; 14^th^ March 2011 to 30^th^ September 2014, the RLTC alone identified call cases for physician team dispatch representing a reasonable sample of the system after reconfiguration.

Other system components remained unchanged.

Cases were abstracted from the NSW Institute of Trauma and Injury Management (ITIM) State trauma registry, Australia, if they met the following inclusion criteria:

- Age < 16 years.
- Incidents within the Sydney coordination area of the NSW Ambulance.
- Injury Severity Score (ISS) > 15 (Abbreviated Injury Scale Score 2005 definitions with 2008 update)
- Acute injury with incident notification occurring via the 000 public access emergency call system.
- Incidents occurred during Period A or B as specified above.

Cases transported to hospitals within the study catchment area by private vehicle were excluded as in the previous study.

The CareFlight P-HEMS screening process utilised all four crew members (consultant grade doctor, paramedic, pilot and aircrewman) rotating hourly to monitor the CAD screens. The RLTC system utilises a paramedic working either solo or paired with a second paramedic monitoring the same screens in a centralised control room. Both systems are fully described in the previous study [8]. After withdrawal of CareFlight P-HEMS access to the CAD system by NSW Ambulance the RLTC system continued to operate at all times when CareFlight P-HEMS were available for response.

### Characteristics of participants and description of materials

During Period A the case identification systems looked for cases that met an agreed set of criteria used by both case identification systems. Criteria were:

All Severe Paediatric Trauma (<16 years) regardless of GCS including:

- Head Injury
- Truncal Trauma
- Limb injury
- Penetrating Injury
- Near drownings
- Burns, especially airway
- Multi Casualty Incidents where a child is likely to be involved

There was no change to these criteria after the withdrawal of CareFlight P-HEMS access to the CAD system, designated Period B. The RLTC did not utilise a standardised interrogation system to assess eligibility against these criteria with paramedics using their clinical judgement to select appropriate cases.

System performance is compared between Periods A & B during CareFlight P-HEMS hours of availability. Therefore the single difference between these two time periods is whether the CareFlight P-HEMS and RLTC case identification systems were operating in parallel or the RLTC system was operating alone. NSW Ambulance physician teams (Greater Sydney Area HEMS, GSA-HEMS) also operated in the catchment area throughout the entirety of Periods A & B and were tasked solely by the RLTC. Patients not identified for physician response all received road paramedic care.

### Statistical methods

The primary outcomes were direct transfer rate to PTC and time from injury to arrival at PTC. Missing total time from injury to arrival at the final hospital was inputted using the median value (4 cases in Period A and three cases in Period B). Data were checked for normality using Shapiro-Wilk’s test. Initially, the differences in patient characteristics and paediatric trauma system performance between the two time periods were compared using appropriate Chi-squared test and Mann Whitney U-test. Kruskal-Wallis test was used to examine the differences in median time from injury to PTC arrival and ISS by the four subgroups of team and period, with *P* value significance adjusted for post-hoc multiple comparisons. Next, the difference-in-differences approach [10] was used to evaluate the change in tasking system on direct transportation to PTC by modelling it as a function of retrieval team care (0 = paramedic 1 = retrieval team), time (0 = May 2008 to 13 Mar 2011, 1 = 14 Mar 2011 to Sept 2014) and an interaction term between retrieval team care and time, adjusted for confounders (ISS and age) using multivariable logistic regression. Adjusted relative risk (RR and 95 % confidence interval (95 % CI) are reported using the results from the multivariable logistic regression to facilitate the interpretation of the difference-in-difference analysis. The coefficient for the interaction term between retrieval team care and time indicates whether retrieval team improved more or less than paramedics from May 2008–13 Mar 2011 (pre) to 14 Mar 2011–Sept 2014 (post). The discrimination and calibration of the model were assessed by estimating a AUROC (95 % CI) and the Hosmer-Lemeshow goodness-fit test, respectively. Statistical analyses were performed using SPSS version 23 (IBM, New York).

## Results

Seventy one cases met the inclusion criteria in Period A (34 months) and 126 cases during Period B (54 months). Characteristics of patients including Intensive Care Unit (ICU) and hospital length of stay during the two time periods are compared in Table 1. Patients were similar apart from a higher median ISS in Period B. Table 2 details the difference between the periods in case identification rates, transport rates to PTCs, time intervals, treating teams and transport modes. The case identification rate halved from Period A (62 %) to Period B (31 %) and time taken to reach a PTC increased from 69 to 97 min (*P* = 0.003). The overtriage rate could be calculated for the CareFlight P-HEMS service only as information on dispatches of the GSA-HEMS to non-severe paediatric trauma was not available to the investigators. In Period A CareFlight P-HEMS was dispatched to 429 paediatric cases of which 36 had ISS > 15 (8.4 %) whereas in Period B this was 423 cases with 27 having an ISS >15 (6.4 %, *P* = 0.26).

**Table 1:** Patient characteristics comparing Period A (May 2008 –March 2011) with Period B (March 2011–September 2014)

	Period A (*n* = 71)	Period B (*n* = 126)	*P* value
Median (IQR) age, years	6 (2–12)	8 (3–13)	0.232
Median (IQR) ISS, all patients • Paramedic patients • Physician patients	22 (17–25) 18 (16–22) 25 (20–29)	25 (19–29) 25 (17–26) 26 (25–31)	0.012 0.009 0.284
Mechanism of injury (n, %)			0.206
Drowning Fall MVA MBA Pedal cyclist Pedestrian Hanging/suffocation Other	12 (16.9) 20 (28.2) 6 (8.5) 1 (1.4) 3 (4.2) 14 (19.7) 2 (2.8) 13 (18.3)	15 (11.9) 42 (33.3) 6 (4.8) 10 (7.9) 5 (4.0) 28 (22.2) 8 (6.3) 12 (9.5)	
ICU admission (n, %)	44 (62.0)	76 (60.3)	0.819
Median (IQR) ICU LOS, days	1 (0–6)	1 (0–3)	0.357
Median (IQR) hospital LOS, days	7 (1–15)	6 (2–19)	0.657

*IQR* interquartile range, *SS* injury severity score, *MVA* motor vehicle accident, *MBA* motor bike accident, *ICU* intensive care unit, *LOS* length of stay

**Table 2:** Case identification rates, transport rates to Paediatric Trauma Centres (PTCs), time intervals, treating teams and transport modes between Period A and Period B

	Period A (*n* = 71)	Period B (*n* = 126)	*P* value
Identified for physician team response (n, %)	44 (62.0)	39 (31.0)	<0.001
Proportion of fatal cases identified for physician team response (proportion, %)	16/16 (100)	14/30 (46.7)	<0.001
Direct transport to PTC (n, %)	47 (66.2)	67 (53.2)	0.076
Definitive care at PTC (n, %)	61 (85.9)	100 (79.4)	0.253
Median (IQR) time from injury to final hospital arrival, minutes	67 (50–97)	82 (51–250)	0.034
Median (IQR) time from injury to PTC arrival for all patients, minutes • Paramedic patients • Physician patients	69 (52–104) 78 (60–291) 61 (50–80)	97 (56–305) 156 (69–341) 67 (54–91)	0.003 0.770 1.000
Treating team (n, %)			<0.001
Road paramedic CareFlight P-HEMS GSA HEMS	31 (43.7) 36 (50.7) 4 (5.6)	91 (72.2) 27 (21.4) 8 (6.3)	
Mode of transport to first hospital (n, %)			
Road ambulance Helicopter	44 (62.0) 27 (38.0)	103 (81.7) 23 (18.3)	0.002

*IQR* interquartile range, *P-HEMS* Physician Staffed Helicopter Emergency Medical Service, *GSA-HEMS* Greater Sydney Area Helicopter Emergency Medical Service

**Fig. 1:**
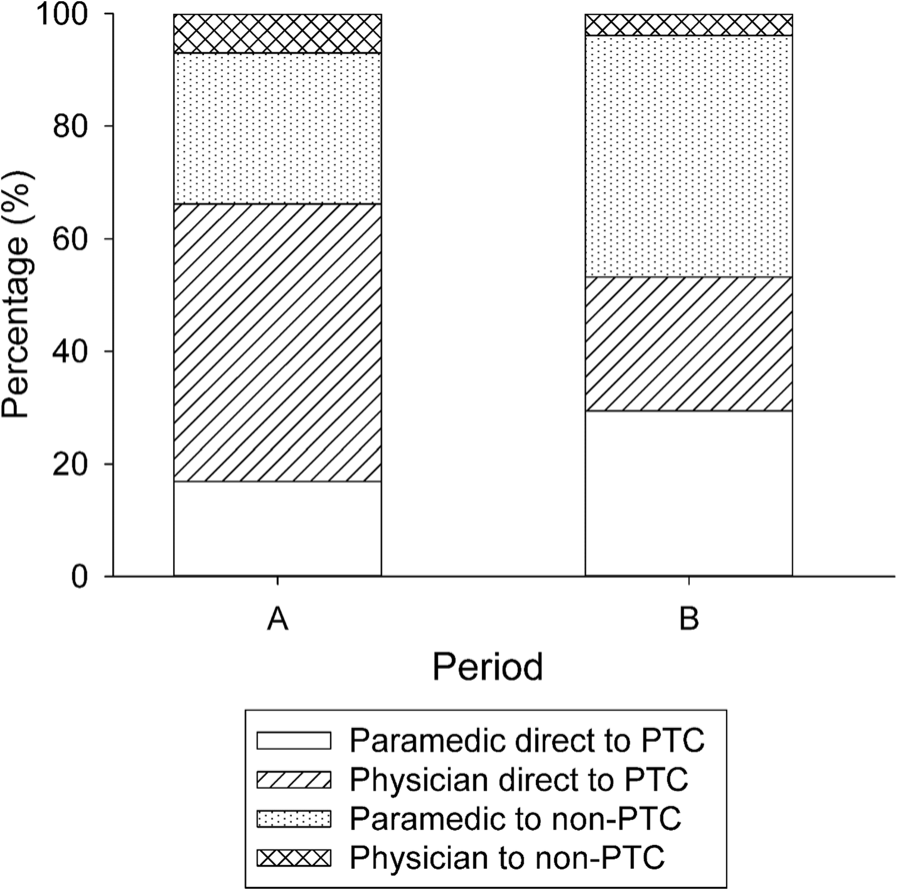
Comparison of the treating team and destination hospital type by time period. The difference between time periods was significant (*P* = 0.001). PTC, Paediatric Trauma Centre

Figure 1 depicts the changes in treating team and first hospital between the two periods. The majority of patients taken by physician teams to non-Paediatric Trauma Centres were 15 year olds transported to an ATC who remained there for definitive care of their injuries.

Table 3 compares the rates of direct transfer by physician and paramedic teams to a PTC between the time periods when controlling for age and ISS using the difference-in-differences methodology. When adjusted for age and ISS, the change from pre to post system (%) in the paramedic group was 2.8 (95 % CI: -17.1 to 22.6) [*P* = 0.785] and in the physician group was -1.2 (95 % CI: -17.6 to 15.1) [*P* = 0.884]. The adjusted relative risk (95 % CI) for the difference in direct to PTC rates between the physician and paramedic groups across time periods was 0.92 (95 % CI: 0.44 to 1.41, *P* = 0.784). The model had acceptable discrimination (AUROC 0.79, 95 % CI: 0.72 to 0.85) and adequate calibration (Hosmer-Lemeshow *P* = 0.834). Physician teams were significantly more likely to transfer a patient directly to a PTC from the incident scene and this did not change across time periods.

**Table 3:** Performance of physician team and paramedic team (reference group) for direct transport to PTC, adjusted for Injury Severity Score and age

	Period A rates (%)			Period B rates (%)			Adjusted change from period A to B system, (%, 95 % CI)^a^	
Indicator	Paramedic	Physician Team	Adjusted RR (95 % CI)	Paramedic	Physician Team	Adjusted RR (95 % CI)	Paramedic	Physician Team
Direct to PTC	12/31 (38.7)	35/40 (87.5)	2.20 (1.39–3.48)	37/91 (40.7)	30/35 (85.7)	2.02 (1.53–2.67)	2.8 (-17.1 to 22.6)	−1.2 (-17.6 to 15.1)

^a^Follow-up percentage minus the baseline percentage

## Discussion

The RLTC case identification system operating in isolation resulted in a fifty percent decrease in the case identification rate for severely injured children, with significant increases in the time taken for arrival at a PTC when compared with a parallel tasking system of CareFlight P-HEMS crew and RLTC working together. As patients in the RLTC only period had higher ISS it might be expected that the rate of physician tasking and of direct transport to a PTC would be higher in this period however the reverse was observed. No children died that were missed for physician dispatch in Period A (only patients that are alive long enough to be transported to hospital are included in the Registry). This suggests that the majority of critically injured children were identified in Period A and that missed dispatch did not have an effect on mortality in this period as all missed cases survived. The CareFlight P-HEMS/RLTC parallel model identified all fatalities compared with only 47 % by the RLTC operating alone.

The time taken to reach a PTC increased by nearly 30 min when the RLTC was operating in isolation, and the rate of helicopter transport halved. All the HEMS in the Sydney area are physician staffed, and failure to identify those critically injured children requiring physician response may have contributed to the lower rate of helicopter transport and subsequent increase in prehospital times. A previous study has also demonstrated significantly higher intervention rates by physician teams in adult patients [11] in our system which is similar to the intervention rate reported in paediatric patients by the CareFlight P-HEMS [12]. Physician teams may therefore have been prepared to transport patients over longer distances as critical procedures have been performed on scene. In the previous study [9] the rate of direct transfer to a PTC was significantly lower when the CareFlight P-HEMS was not available. In this study, during CareFlight P-HEMS operational hours, the rate of direct transfer was lower when the RLTC was tasking in isolation.

The difference-in-difference analysis indicated that the rate at which either physician or paramedic teams transported directly to a PTC did not change across time periods suggesting that this is independent of the tasking system, and is likely to be related to the clinical knowledge and skill set of the respective treating teams, and the transport options available to them in our system. Physician treated patients had little change in time to PTC and little change in ISS between periods whereas for paramedic treated patients in Period B the time increased and the ISS was significantly higher. This might suggest more time on scene with more severely injured patients although the time difference by treating team did not reach significance. Regardless the physician treated patients had similar ISS in both time periods to the paramedic treated patients in Period B but the times to PTC were much shorter (*P* = 0.003) supporting physician dispatch as the system policy.

Definitive proof that physician team response to severe trauma improves outcome is not available. Nonetheless, physician staffed teams were adopted as a standard by NSW Ambulance in 2008 [13]. This was supported in 2013 by the NSW Ministry of Health Reform Plan for Aeromedical (Rotary Wing) Retrieval Services [14], which specified a move to a standard physician/paramedic model for all HEMS prehospital missions. The utility of such a system however depends on accurate case identification processes to ensure that the patients most likely to benefit are recognised. This study demonstrates that the withdrawal of CareFlight P-HEMS access to the NSW Ambulance case identification system resulted in a significant deterioration in system performance with a much lower proportion of severely injured children now receiving physician prehospital care and the time to arrival in a PTC increasing significantly.

P-HEMS crew guided tasking offers several potential advantages compared with a paramedic dispatching from a centralised coordination centre which have already been discussed in the original study [9]. A recent study has also indicated that telephone interrogation of the emergency caller by a flight paramedic is as accurate as direct assessment by on-scene ground ambulance crews [15]. The CareFlight P-HEMS tasking system also made extensive use of caller interrogation which may have contributed to the higher identification rate observed in Period A. Utilising HEMS paramedics in the control room may be an alternative approach to the parallel identification system studied here. For example London HEMS demonstrated a significant improvement in appropriate utilisation after a HEMS paramedic was rotated into the central dispatch centre [16]. This model however does not allow parallel processing by clinical and aviation crew where the pilot can be preparing for engine start whilst caller interrogation by the clinical crew confirms case appropriateness. Published data indicates that the CareFlight P-HEMS dispatch model is airborne approximately 4 min faster than the London HEMS system [11, 15]. The London model also requires an increase in the size of the HEMS paramedic staffing pool with potential safety implications due to decreased team member familiarity [17]. Cost may also be greater - the London HEMS model was the original plan for the CareFlight P-HEMS but the internet link version was favoured as it saved two paramedic salaries.

It appears that the common factor in the CareFlight P-HEMS and the London HEMS systems is that clinicians from the HEMS service are directly involved in the case identification process, in one case via internet link from the HEMS base and the other physically located in the control room. Personnel who are actively involved in the HEMS may be the best placed to determine the suitability of cases as they inherently understand the operational and clinical capability of the service. It is notable that in the CareFlight P-HEMS system some of the screening was performed by the pilot and aircrewman. Although cases identified by these disciplines were always ratified by the doctor or paramedic, this suggests that specialist knowledge of the HEMS capability may be a vital element in accurate tasking that cannot be replaced by general clinical background alone.

Another alternative is to directly utilise the Medical Priority Dispatch System for helicopter dispatch but this was found to increase the over-triage rate when introduced in Austria [18]. Centralised dispatch coordination can however offer significant benefits enabling prioritisation between competing tasks and producing the most efficient overall use of resources. Notably the P-HEMS case identification system for paediatric patients in this study was not a dispatch system but a case identification system only. Response was only activated for identified paediatric cases after consultation with and authorisation by the RLTC. This resulted in no unintended parallel responses to paediatric patients by the study P-HEMS service and other physician staffed services for the 3 years that the two identification systems operated in parallel although in certain situations a parallel response was deliberately initiated to suspected multiple casualty incidents. The parallel identification system appears to offer significant benefits in case recognition, whilst requiring RLTC authorisation for response preserves the safety and efficiency benefits of a centralised dispatch system. During the Head Injury Retrieval Trial in Sydney the study P-HEMS service did not require central authorisation for response to adult patients and parallel responses by two physician teams to the same patient were common [11]. This was an artificial situation produced by conduct of a randomised controlled trial. However the system used for paediatric case identification outside of the trial demonstrates a model that effectively combines the benefits of parallel case identification systems with centralised tasking control.

There are limitations in this study. While before and after studies such as the one reported herein are used frequently in injury research, they do not take into account temporal changes that may be occurring independent of the intervention. However, the authors are not aware of any system changes to either dispatch processes or the general EMS response system that occurred between the two periods apart from the case identification system. Some cases were excluded due to missing data regarding the mode of transport to the first hospital. However, all these cases initially went to non-PTCs and were subsequently transferred, thus their inclusion would only have decreased the overall proportion of patients taken directly to a PTC, and increased the total time to PTC arrival. The joint effect would have been to increase the differences observed in the present study, again favouring a parallel case identification system. Additionally we were unable to calculate sensitivity and specificity of the systems as the data on total physician team taskings was not available. We have calculated the over-triage data for one of the physician services, although it seems unlikely that dispatch accuracy would vary depending on the physician team to whom the task was allocated.

## Conclusion

Implementation of a case identification system that relied solely on paramedic Rapid Launch Trauma Co-ordinators identifying severely injured children resulted in significant negative effects on the paediatric pre-hospital system compared with a case identification system which utilised a P-HEMS crew screening in parallel. Halving of the case identification rate with an increase of 28 min to arrival in a PTC for severely injured children has the potential to affect outcomes. Physician staffed pre-hospital teams were more likely to transfer seriously injured children directly to a PTC compared with paramedic only teams, although the observed decrease between time periods in the overall rate of direct transport to a PTC was not significant. The results of this study suggest that the case identification system can be improved and options such as reintroducing the Careflight P-HEMS parallel tasking system or rotating a HEMS paramedic into the control room should be considered. Accurate case identification results in more capable clinical teams attending the incident scene and shorter overall times to PTC arrival which may potentially affect outcomes.

## Abbreviations

ATC, Adult Trauma Centre; CAD, Computer Assisted Dispatch; GCS, Glasgow Coma Scale; GSA-HEMS, Greater Sydney Area Helicopter Emergency Medical Service; ISS, Injury Severity Scale; ITIM, Institute of Trauma and Injury Management ; NSW, New South Wales; RLTC, Rapid Launch Trauma Coordinator; P-HEMS, Physician staffed Helicopter Emergency Medical Service; PTC, Paediatric Trauma Centre
